# Gesticulation in individuals with at risk mental states for psychosis

**DOI:** 10.1038/s41537-023-00360-1

**Published:** 2023-05-09

**Authors:** Ana Caroline Lopes-Rocha, Willian Henrique de Paula Ramos, Felipe Argolo, João Medrado Gondim, Natalia Bezerra Mota, Julio Cesar Andrade, Andrea Fontes Jafet, Matheus Wanderley de Medeiros, Mauricio Henriques Serpa, Guillermo Cecchi, Anderson Ara, Wagner Farid Gattaz, Cheryl Mary Corcoran, Alexandre Andrade Loch

**Affiliations:** 1 Laboratorio de Neurociencias (LIM 27), Instituto de Psiquiatria, Hospital das Clinicas HCFMUSP, Faculdade de Medicina, Universidade de Sao Paulo, Sao Paulo, SP Brazil; 2 Statistics Department, Federal University of Paraná, Curitiba, PR Brazil; 3 Instituto de Computação, Universidade Federal da Bahia, Salvador, BA Brazil; 4 Instituto de Psiquiatria (IPUB), Departamento de Psiquiatria e Medicina Legal, Universidade Federal do Rio de Janeiro (UFRJ), Rio de Janeiro, Brazil; 5 Research department at Motrix Lab – Motrix, Rio de Janeiro, Brazil; 6 Laboratorio de Neuroimagem em Psiquiatria (LIM 21), Instituto de Psiquiatria, Hospital das Clinicas HCFMUSP, Faculdade de Medicina, Universidade de Sao Paulo, Sao Paulo, SP Brazil; 7 Instituto Nacional de Biomarcadores em Neuropsiquiatria (INBION), Conselho Nacional de Desenvolvimento Científico e Tecnológico, São Paulo, Brazil; 8 IBM T.J. Watson Research Center, Yorktown Heights, NY USA; 9 Icahn School of Medicine at Mount Sinai, New York, NY USA; 10 James J. Peters VA Medical Center, Bronx, NY USA

**Keywords:** Biomarkers, Psychosis, Schizophrenia

## Abstract

Nonverbal communication (NVC) is a complex behavior that involves different modalities that are impaired in the schizophrenia spectrum, including gesticulation. However, there are few studies that evaluate it in individuals with at-risk mental states (ARMS) for psychosis, mostly in developed countries. Given our prior findings of reduced movement during speech seen in Brazilian individuals with ARMS, we now aim to determine if this can be accounted for by reduced gesticulation behavior. Fifty-six medication-naïve ARMS and 64 healthy controls were filmed during speech tasks. The frequency of specifically coded gestures across four categories (and self-stimulatory behaviors) were compared between groups and tested for correlations with prodromal symptoms of the Structured Interview for Prodromal Syndromes (SIPS) and with the variables previously published. ARMS individuals showed a reduction in one gesture category, but it did not survive Bonferroni’s correction. Gesture frequency was negatively correlated with prodromal symptoms and positively correlated with the variables of the amount of movement previously analyzed. The lack of significant differences between ARMS and control contradicts literature findings in other cultural context, in which a reduction is usually seen in at-risk individuals. However, gesture frequency might be a visual proxy of prodromal symptoms, and of other movement abnormalities. Results show the importance of analyzing NVC in ARMS and of considering different cultural and sociodemographic contexts in the search for markers of these states.

## Introduction

Nonverbal communication (NVC) is defined as interpersonal communicative behavior that does not involve spoken words. Instead, it involves motor domains such as facial expressions, voice modulation, body posture, and gestures. Gestures, more specifically, play an important role in communication, countering difficult conditions, as in situations with high noise, enhancing comprehension, and providing a link between action and mental representation^1–3^. Impairments in gesture are common in individuals with schizophrenia^4–7^, and are visible signs which translate the disease’s physiopathology, for they are associated with motor abnormalities, dysfunction in the frontal lobe, and negative symptoms^7–9^. They can be investigated mainly through their perception, interpretation, or performance^10^. During clinical evaluation, abnormal gestures are frequently seen as part of blunted affect expression^11–13^, and, in addition to performing, gesture impairment in schizophrenia also includes deficits in gesture recognition, tool use, and poor nonverbal social perception^6,7^.

Impairments in gestures are also seen in early-stage psychosis, including schizotypal individuals and people with “at risk mental states” (ARMS) for psychosis—also known as clinical high risk or CHR. The use of more retrieval gestures—those used during pauses in speech^14^—fewer gesture performance^15^, a negative correlation for certain gestures categories with positive symptoms^15^, semantic incongruence between gesture and speech, an association between this mismatch and negative symptoms^14^, and abnormal gesture perception^16^ are some examples of gestures performance impairments in these individuals. Retrospective parent reports also showed that at-risk individuals exhibited more gestural communication deficits during early childhood and that deficits were associated with future worse functioning and more severe negative symptoms in such subjects^17^—replicating the relationship between gesture impairment and worse outcomes in schizophrenia^9,18^.

Apart from the above-mentioned works, published studies on gesture abnormalities—and movement abnormalities in general—in ARMS subjects are still scarce, though. Some of them used the open-source software Motion Energy Analysis (MEA)^19^. MEA is a software that automatically quantifies movement by capturing differences in grayscale pixels frame by frame in a predefined region of interest. We have published an analysis similar to one performed by Dean et al. (2018)^20^ to assess the amount of general movement in ARMS during speech tasks (see more information in Lopes-Rocha et al., 2022). A reduced total movement frequency and increased movement variability was seen for ARMS subjects, and these variables were significantly correlated with positive and negative symptoms^21^. However, it was not possible to infer if MEA findings were due to gesture deficits or not, as the software only “sees” general movement and is not capable of discriminating gestures.

To summarize, further studies need to investigate if movement abnormalities—and gestures abnormalities, more specifically—seen in schizophrenia are also present in preclinical stages of the disorder (i.e., ARMS subjects), and if they are related to specific symptom domains at this early stage. Available data on this issue is scarce, and most of the published studies are based on help-seeking samples, which encompasses potential selection biases. The detection of gestural abnormalities and/or their relationship with specific symptom domains would add objective measures to the assessment of ARMS subjects, as well as enable the investigation of potential early biological underpinnings of such abnormalities and outcome prediction.

As such, the present study aimed to analyze gesture performance described in 56 medication-naïve individuals identified as ARMS and 64 healthy controls. For this, audiovisual data obtained during two different speech tasks—subject overview (SO) and memory reports (MR)—were manually analyzed by a coder fully blind to the participant group, and the frequency of four gestures (iconic, deictic, metaphoric, and beats) and self-stimulatory behaviors during these videos were obtained. Given the reduced movement obtained in our previous study and the gestures deficits observed in other studies, we hypothesized that: (1) ARMS individuals perform fewer gestures compared to healthy controls; (2) there is a negative correlation of gesture performance with total positive and negative symptoms obtained by Structured Interview for Prodromal Syndromes (SIPS), (3) gesticulation is associated with the energy of movement (MEA variables published in our previous study). We also opted to explore the relationship between gestures and disorganization and the general symptoms dimension.

## Results

Sociodemographic data did not differ between ARMS and controls (Table 1). A significant difference between the ARMS and control groups was observed for deictic gestures in SO video (Table 2); however, significance did not survive Bonferroni correction for multiple comparisons (*p* > 0.006). No significant differences were seen for other gesture categories.

**Table 1:** Sociodemographic of ARMS and control groups.

		ARMS (*n* = 56)	Control (*n* = 64)	Statistic	*ρ*
Age (years)^*^		28.1 ± 4.4	27.3 ± 4.8	*t*(117) = 0.98	0.33
Gender^#^	Female	37 (66.1)	44 (68.8)	χ^2^ = 0.10, df = 1	0.75
	Male	19 (33.9)	20 (31.3)		
Education (level)^#^	Incomplete or complete high school	19 (35.2)	18 (28.1)	χ^2^ = 0.73, df = 2	0.69
	Incomplete or complete undergraduate degree	31 (57.4)	40 (62.5)		
	Incomplete or Complete Graduate Degree	4 (7.4)	6 (9.4)		

*Mean ± SD.

^#^*N* (%), two participants in the ARMS group did not answer the question about education.

**Table 2:** Gesticulation and self-stimulatory movements in ARMS and control.

	Subject overview (SO)			Memory report (MR)		
	Control	ARMS	*p*value	Control	ARMS	*p*value
Iconic^a^	2.9 (5.0)	2.6 (4.1)	0.32	7.12 (12.0)	11 (17.5)	0.75
Metaphoric^a^	18.6 (20.7)	15.3 (16.9)	0.24	26.4 (29.8)	23.6 (30.5)	0.20
Beats^a^	189 (171)	163 (203)	0.079	165 (147)	135 (134)	0.15
Deitics^a^	65.2 (43.4)	57.7 (50.6)	0.048^*^	62.6 (55.7)	52.7 (42.4)	0.24
Self-stimulating^b^	24.4 (26.1)	24.7 (22.7)	0.27	26.6 (28.6)	27.2 (30.2)	0.55

Mean (SD) × 10^-^³. *p*value obtained by Mann–Whitney *U*-test.

^a^Alternative hypothesis ARMS < Control.

^b^Alternative hypothesis ARMS > Control.

Our second hypothesis—a negative correlation between gesture and positive and negative symptoms—was confirmed, as shown in Table 3. Using Kendall’s Tau-B correlation, deictic gestures made during SO and metaphoric gestures made during the MR task were inversely correlated with total negative symptoms. Total positive symptoms were inversely correlated with beats and deictic gestures in SO. Using the Generalized Linear Model by negative binomial distribution (GLM-NB), deictic gestures in both videos (SO: *z* = −2.548, *p* = 0.011; MR: *z* = −2.145, *p* = 0.032) and the metaphoric gestures in MR (*z* = −2.626, *p* = 0.009) were significant estimation parameters for total negative symptoms. For positive symptoms, GLM only showed a marginal significance for deictic in SO (*z* = −1.884, *p* = 0.059).

**Table 3:** Correlation between SIPS symptoms domains and gesticulation and self-stimulating movements.

Video	Category	Negative^a^	Positive^a^	Disorganization^b^	General^b^
		Tau-B	Tau-B	Tau-B	Tau-B
SO	Iconic	0.056	0.069	0.084	0.009
	Metaphoric	−0.045	−0.040	0.017	−0.074
	Beats	−0.089	−0.123^*^	−0.049	−0.020
	Deitics	−0.209^***^	−0.193^**^	−0.127	−0.117
	Self-stimulating^c^	−0.075	0.056	−0.092	−0.009
MR	Iconic	0.043	0.018	0.076	−0.042
	Metaphoric	−0.175^**^	−0.048	−0.033	−**0.127**
	Beats	−0.086	−0.077	−0.045	−0.060
	Deitics	−**0.089**	−0.090	−**0.106**	−0.026
	Self-stimulating^c^	−0.112	−0.048	−0.058	−0.071

**Bold**: significant as parameters for estimating symptoms using a generalized linear model by a negative binomial distribution.

*p < .05, **p < .01, ***p < .001, by Kendall’s Tau-B Correlation.

^a^Alternative hypothesis: correlated negatively.

^b^Alternative hypothesis: correlated.

^c^Alternative hypothesis: correlated positively with negative and positive symptoms.

In our exploratory investigation (Table 3), no significant correlation between gestures categories or self-stimulatory movement was seen for other SIPS categories using Kendall’s Tau-B, but using GLM-NB, we found that total general symptoms was estimated by metaphoric in MR (*z* = −2.337, *p* = 0.019) and total disorganization tended to be estimated by deictic in MR (*z* = −1.898, *p* = 0.057).

Concerning our last hypothesis, on the existence of an association between the MEA variables and those collected here, it was verified as shown in Table 4. In general, positive correlations were seen between mean amplitude and frequency of head and torso and gestures categories, while negative correlations were seen between gestures and coefficient of variability.

**Table 4:** Correlation between movement variables collected with MEA, gesticulation, and self-stimulatory movements.

			Subject overview (SO)					Memory report (MR)				
			Iconic	Metaphoric	Beat	Deictic	Self-stimulatory	Iconic	Metaphoric	Beat	Deictic	Self-stimulatory
SO	Head	Mean amplitude	0.18^*^	0.2^*^	0.21^**^	0.25^**^	−0.02	0.19^*^	0.06	0.22^**^	0.09	0.04
		Frequency	0.14	0.08	0.14	0.08	−0.05	0.08	−0.03	0.07	0.1	0
		Coefficient of variability	−0.11	−0.23^**^	−0.28^***^	−0.23^**^	0.01	−0.13	−0.01	−0.21^**^	−0.13	−0.03
	Torso	Mean amplitude	0.17	0.38^***^	0.53^***^	0.26^**^	0.19^*^	0.16	0.17	0.38^***^	0.15	0.11
		Frequency	0.13	0.21^*^	0.32^***^	0.12	0.1	−0.01	0.02	0.16^*^	0.05	0
		Coefficient of variability	−0.13	−0.29^***^	−0.55^***^	−0.18^*^	−0.25^**^	−0.09	−0.09	−0.32^***^	−0.16	−0.14
MR	Head	Mean amplitude	0.07	0.14	0.19^*^	0.28^***^	−0.02	0.24^*^	0.1	0.24^**^	0.17^*^	0.08
		Frequency	0.06	−0.05	−0.03	0.18^*^	−0.04	0.19^*^	0.08	0.11	−0.01	0.17^*^
		Coefficient of variability	−0.19^*^	−0.11	−0.11	−0.2^*^	0.02	−0.17	−0.15	−0.2^*^	−0.07	−0.03
	Torso	Mean amplitude	0.04	0.19^*.^	0.36^***^	0.32^***^	0.12	0.23^*^	0.2^*^	0.43^***^	0.13	0.22^*^
		Frequency	0.06	0.02	0.17^*^	0.17^*^	0.06	−0.03	−0.02	0.23^**^	0.11	0.18^*^
		Coefficient of variability	−0.08	−0.18^*^	−0.31^***^	−0.21^*^	−0.21^*^	−0.14	−0.24^**^	−0.41^***^	−0.05	−0.28^***^

*p < .05, **p < .01, ***p < .001, by Kendall’s Tau-B correlation. Alternative hypothesis: correlated.

## Discussion

In our study of gesticulation in medication-naïve ARMS subjects, less deictic gestures in ARMS were observed compared with controls. Although this result did not survive Bonferroni’s correction, this gesture category was correlated with more negative and positive symptoms. Besides, negative symptoms correlated with metaphoric, and positive symptoms with beats gestures. At last, less gesticulation was significantly correlated with a reduced total amount of movement and higher variability of movement.

Our study contrasts with the previous one by Mittal et al. (2006), who reported reduced gesture rates and increased self-stimulatory movements in 20 SPD individuals^15^. Here, ARMS and controls differed only for deictic gestures, but this result did not survive Bonferroni’s correction, and these differences might be related to sample differences. For instance, age impacts on gestural performance—their participants were aged 12 to 18 while ours were between 18 and 25 years^22^. Also, our study analyzed these gesture categories in a Brazilian sample of ARMS individuals—and culture also mediates gesture expression^22–24^. In addition, methodological differences may also have influenced these results: while our analysis was performed using two different free-speech tasks, they filmed the first 30 min of a structured diagnostic interview. Our objective was to use tasks that allowed the assessment of gesticulation in a closer way to those performed in spontaneous interactions. Aiming for more naturalistic data, we tried, thus, to reduce the tension that participants may feel during diagnostic interviews that could end up accentuating gesture differences between ARMS and control subjects.

Poor gesture performance is also observed in schizophrenia. In addition to speaking less, 20 patients diagnosed with schizophrenia also made less use of hand gestures during interaction with healthy controls to discuss a moral dilemma^4^. Reduced gestures were also seen for 25 drug-free schizophrenia patients in an ethological study^25^. Impairments in imitation and pantomime hand gestures are pointed out in several studies^26–30^. For example, using the Test of Upper Limb Apraxia (TULIA) to assess these categories in 30 schizophrenia patients, it was observed that 66.7% presented deficits^26^. However, increasing this sample to 89 patients, only 52% presented gesture impairments^8^.

It is possible that these differences found in schizophrenia studies are related to deficits’ evolving during the course of the disease. For instance, a study conducted by Stegmayer et al. (2016) showed that 14 multiple-episode patients presented severe gesture deficits compared to 14 first-episode subjects^5^. On the other hand, Lavelle et al. (2014) interestingly did not find significant differences between schizophrenia patients and psychiatrists regarding gestural movements. Authors pointed to the possibility that these patients might be divided into two different categories of nonverbal behavior: one prosocial to take part in interactions and one to avoid social interactions^31^. Given that here we are investigating deficits in ARMS individuals, it is possible that the lack of differences for gesture categories might be related to the actual stage that they are or, moreover, that they are part of the group with more prosocial behavior. However, this is speculative, and it is necessary to investigate these variables longitudinally, after the transition to psychosis.

Regarding the findings for the correlation between gestures and SIPS symptoms, there are few studies that evaluate it in ARMS condition. Mittal et al. (2006) found a negative correlation between iconic gestures and positive symptoms, but no correlations for other gestures or for negative symptoms^15^. Osborne et al. (2017) also did not find any association between beat gestures and SIPS’ positive or negative symptoms^32^. Although our results are different from theirs, they agree with findings for schizophrenia patients where gesture deficits are mostly associated with negative symptoms^4,5,8,13^. In schizophrenia, gesture performance was also associated with the positive symptom dimension^30^. Walther et al. (2015) found a negative association between gesture performance and positive symptoms in a study conducted with 46 patients with schizophrenia, schizoaffective or schizophreniform disorder, and 44 healthy controls^7^. We also found a negative correlation between total negative symptoms and some categories (deictic and metaphoric), and between total positive symptoms and deictic and beats gestures. Also, deictic gestures showed to be a significant estimate parameter for negative and disorganization symptoms, and metaphoric gestures for negative and general symptoms. In summary, our findings present convergent validity, in the sense that our behavioral measures are associated with the existing ground truth of clinical ratings of emotional expression.

Gesture deficits in schizophrenia also showed to be an important predictive factor for disease outcome. In a 6-month follow-up study, schizophrenia patients with gestural impairments in baseline showed a higher level of overall negative symptoms after the period when compared to those without such impairments^9^. Gesturing involves different processing modalities and the coordination of different brain areas^15,33^. In general, the impairments in schizophrenia are linked to frontal lobe dysfunctions, motor abnormalities, and working memory deficits^7,34,35^. For example, in patients with impairments in gesture production, a reduced cortical thickness in eight ROIs, including precentral gyrus, insula, inferior, and superior parietal lobe^36^. However, studies also showed that different gestural categories might be more related to specific regions^33,35,37^. So, it is possible that the observable gesture performance seen in consonance with SIPS symptoms represents early brain dysfunction in ARMS. Future studies should further explore this hypothesis.

At last, we observed that gesture performance was related to the energy of movement—assessed in a previous work of ours. In these findings, a reduced movement and increased variability of movements in ARMS was observed with MEA^21^. Given that MEA only measures how much the person moved and not the type of movement made, in the present study, we were able to see that gesticulation was positively correlated with the amount of movement and negatively correlated with movement variability. Thus, the reduced movement obtained with MEA is largely attributed to the use of gestures, even though differences within these categories have not been observed here. It is important to highlight, however, that other movement variables beyond the gesture itself, such as postural sway^38,39^, might also be contributing to the significant differences found in MEA, but no other specific movement parameter was obtained to verify this hypothesis. In addition, these findings show the importance of both manual and automated analysis, which are complementary to each other in the analysis of NVC in ARMS.

It is also important to consider that most studies that evaluate gesture impairment focus on performance errors through elicitation tasks, such as TULIA. Our focus here was to use a different approach, focusing on the use of gestures in a more naturalistic way, in how this sample express itself using this type of communication when compared with healthy controls and not how much they fail in their use of gesticulation, bringing new additions to the field. Also, despite the lack of differences between the groups, the correlation between symptoms and MEA variables shows that gestures might be related to the deficits found in them. Thus, it is possible that gesticulation differences within the ARMS group itself have impacted this result, and it is a hypothesis that we will be able to test soon, after their outcome.

Our study has some limitations. First, we worked with a limited sample size. However, our sample was formed by non-helping-seeking medication-naïve individuals and findings were consistent with previously published studies in ARMS^40,41^. Also, no differences in demographic data were seen between them and the control group, increasing its reliability. Second, a large number of statistical tests were made. We tried to minimize type I error by using the General Linear Model to infer individual symptoms from gestures, but the possibility of statistical bias cannot be ruled out. Third, gesticulation was assessed manually. Considering that we have already used MEA to analyze the total amount of movement in this sample, it was necessary to understand the type of movement performed. For this, the evaluation of different gestural categories depended on the analysis of the semantic context of the verbal component associated with the co-speech gesture and automatic analysis does not currently allow this classification. Thus, this limitation is intrinsic to the analysis performed.

Summarizing, the results we were able to show here are important features to be considered in the visual phenotyping of at-risk individuals. Our findings raise several possibilities, such as the use of gestures analysis in videos collected by naturalistic ways to improve the classification of symptom severity in ARMS individuals. Considering that there are few studies carried out in developing countries^42^, as stated before, our results also bring important data that should be used for cross-cultural analysis between different ARMS samples, to assess culture-specific as well as universal parameters on gestural impairments across the psychosis continuum. Future directions point us to the assessment of gesture evolution in these at-risk subjects over time, following up with them to see if any baseline gestural feature has predictive power on the outcome, for instance. Also, multimodal analysis—e.g., brain imaging—should be planned to test the use of gesture impairment as a potential endophenotype for schizophrenia spectrum disorders.

## Methods

### Sample and procedures—participants

With the aim of following up with ARMS individuals, the Subclinical Symptoms and Prodromal Psychosis (SSAPP) project^43^ sampled a population-based cohort of 7000 individuals aged between 18 and 36 years. This cohort was built through three waves of general population screenings, in 2016–2017, 2021, and 2022, and details on the procedures can be found elsewhere^43,44^. The present study capitalizes on data obtained in the second and third waves of recruitment. Briefly, subjects were screened with the Prodromal Questionnaire-Brief version (PQ-16), a 16-item self-reported questionnaire to screen for ARMS^45^, and the Basic Symptoms scale (BS), a 9-item scale based on self-experienced disturbances in perception and cognition^46,47^. Those with scores>10 on the sum of PQ-16 + BS were called to a face-to-face interview at the Institute of Psychiatry, University of Sao Paulo, Brazil, for assessment of ARMS status, following previously published recommendations^46^.

ARMS status was assessed with SIPS^48,49^, which diagnoses three prodromal syndromes for psychosis (Brief Intermittent Psychotic Symptom syndrome—BIPS, Genetic Risk and Deterioration syndrome—GRD, and Attenuated Psychosis Syndrome—APS) and the Structured Interview for DSM-5 diagnosis (SCID-5)^50^—evaluating DSM-5 disorders. Among those screened, we identified 56 individuals who met ARMS criteria and 64 healthy controls, all participants were medication-naïve.

### Audiovisual acquisition

Participants were positioned in front of a mobile phone, which was on steady support, that recorded the audiovisual file of two speech task protocols: subject overview (SO) and memory report (MR). SO consisted of the SIPS “subject overview” section, which entailed a request to talk about their childhood and relationship with their parents and it was collected at the start of the interview. MR was based on Mota’s paradigm^51,52^, requesting oral memory reports of a recent dream, an old dream, and short-term memory reports based on three positively affective pictures (a baby, a puppy, and a dessert), collected at the end of the interview. In cases when participants did not remember a dream, a description of the prior day was solicited. The use of these two different tasks was aimed to assess a freer and spontaneous gesticulation in different moments of the clinical interview, and therefore, contrary to other studies, we preferred not to analyze videos of the diagnostic interview itself. All videos were stored in a digital privacy-compliant cloud immediately after collection and deleted from the mobile phones.

### Gesticulation and self-stimulatory movements analysis

The gesture analysis adopted was based on the article of Mittal et al. (2006), considering self-stimulatory movements and four categories of gestures: iconic, metaphors, beats, and deictics^15^. Iconic gestures refer to what is being said in a concrete way; e.g., someone talking about a ball and making a round form with their hands. Metaphoric gestures represent verbal speech through abstract ideas—someone raises their hand while saying the music is too loud. Beat gestures are rhythmic movements made during the speech that do not represent the meaning of what is being said, as up and down movements with the hand during speech. Deictic gestures refer to both the abstract and the concrete domains—pointing with fingers to refer to someone that is not present at the moment^53^. All these actions require some degree of abstraction and motor coordination, which are typically impaired in schizophrenia spectrum disorders^1–3,53–55^. Besides gestures, another type of motor behavior commonly performed during speeches are self-stimulatory movements. They consist of repetitive movements made whether the subject is speaking or not, which do not perform a social function^56^—as a movement directed at the person, such as touching the hair, hands, or ring^15^.

All 240 videos were encoded by the same researcher, fully blind to individuals’ diagnosis. Before starting the coding, the researcher dedicated herself to studying the methodology applied in the base study and have trained in pilot videos. The four gesture categories were analyzed based on the frequency of each one, given by the total number of actions divided by the speech time. The frequency of self-stimulation movements was given by the total number of actions divided by the total video time, since this behavior does not depend on speech to be classified.

### Symptoms

Ratings of positive and negative symptoms collected by the SIPS^48^ were used to test for hypothesized associations with gestures and self-stimulatory movements in our sample. The sum of total positive and negative symptoms were considered to test the hypothesis of a negative correlation between them and the four categories of gestures (iconic, metaphoric, deictics, and beats). Total disorganization and general symptom scores were also used to explore a possible relationship with gestures.

### Association with variables collected with motion energy analysis

Our sample collected here overlaps the sample of our previous publication and in this way, we seek to verify the hypothesis of the existence of an association between the gesticulation variables collected here and previously published data. Thus, we used ratings of mean amplitude, frequency, and coefficient of variability of movements of the head and torso of 32 ARMS and 46 healthy controls collected by the open-source software MEA to perform correlation with the presenting data. MEA detects movement using a frame-differencing method that evaluates differences in grayscale pixels frame by frame and quantifies it as energy motion^19,57^. More information about the collection of this data and the results obtained can be found in our article^21^.

### Statistical analysis

All statistical tests were performed in SPSS version 25 for Mac. The distribution of each movement variable (gestures categories and self-stimulatory movements) in each video task (SO and MR) was evaluated by the Shapiro–Wilk test for normality. Either Student’s *t*-test or non-parametric Mann–Whitney *U*-test were performed to verify differences between ARMS and controls. Kendall’s tau-B correlation coefficient was obtained for the correlation between SIPS symptoms and gesticulation. After analyzing the data distribution, the one who did better was the Generalized Linear Model by negative binomial distribution (GLM-NB) and it was performed to verify the prediction for symptoms by the gestures and self-stimulatory variables. Lastly, to verify the association between MEA variables for both head and torso (mean amplitude, frequency, and coefficient of variability), gesticulation, and self-stimulatory movements, we also used Kendall’s tau-B correlation.

### Ethics and inclusion statement

All the participants provided written informed consent for research procedures, and the research was approved by the research ethics committees: Comissão Nacional de Ética em Pesquisa (No. 53536816.0.0000.0065) and Comitê de Ética em Pesquisa da Faculdade de Medicina da Universidade de São Paulo (No. 36510820.3.0000.0068). Privacy protection regarding the recorded material was granted according to Brazilian data protection compliance standards (Lei Geral de Proteção de Dados, LGPD; https://www.lgpdbrasil.com.br) by means of current encryption protocols in the backend database and over the remote communications (SSL). The research included local researchers in the process of study design, implementation, and data ownership, with outside collaboration only for the writing of the manuscript.

## Data Availability

Given the sensitivity of video of individuals' faces, video data are not available. However, gestures and demographic variables are available in Excel format. Also, given ethical restrictions related to the participants, the data are available under request from the author A.A.L.
